# Atenolol Reduces *Leishmania major*-Induced Hyperalgesia and TNF-α Without Affecting IL-1β or Keratinocyte Derived Chemokines (KC)

**DOI:** 10.3389/fphar.2016.00022

**Published:** 2016-02-15

**Authors:** Marc C. Karam, Rana Merckbawi, Sara Salman, Ali Mobasheri

**Affiliations:** ^1^Department of Biology, University of BalamandKourah, Lebanon; ^2^Department of Veterinary Preclinical Sciences, School of Veterinary Medicine, Faculty of Health and Medical Sciences, University of SurreyGuildford, UK; ^3^Center of Excellence in Genomic Medicine Research, King Fahd Medical Research Center, Faculty of Applied Medical Sciences, King Abdulaziz UniversityJeddah, Saudi Arabia

**Keywords:** atenolol, *Leishmania major*, inflammation, hyperalgesia, TNF-αlpha, prostanoids, sympathetic amines

## Abstract

Infection with a high dose of the intracellular parasitic protozoan *Leishmania major* induces a sustained hyperalgesia in susceptible BALB/c mice accompanied by up-regulation of the pro-inflammatory cytokines IL-1β and IL-6. Interleukin-13 (IL-13) has been shown to reduce this hyperalgesia (despite increased levels of IL-6) and the levels of IL-1β during and after the treatment period. These findings favor the cytokine cascade leading to the production of sympathetic amines (involving TNF-α and KC) over prostaglandins (involving IL-lβ and IL-6) as the final mediators of hyperalgesia. The aim of this study was to investigate the effect of daily treatment with the β-blockers atenolol on *L. major*-induced inflammation in mice with respect to hyperalgesia as well as the levels of TNF-α and KC (the analog of IL-8 in mice). Our data demonstrates that atenolol is able to reduce the *L. major* induced sustained peripheral hyperalgesia, which does not seem to involve a direct role for neither IL-lβ nor KC. Moreover, our results show that TNF-α may play a pivotal and direct role in sensitizing the peripheral nerve endings (nociceptors) since its level was reduced during the period of atenolol treatment, which correlates well with the reduction of the observed peripheral, but not central, hyperalgesia. These findings contribute to a better understanding of the cytokine cascade leading to hyperalgesia and may lead to the development of new and more efficient medications for many types of pain.

## Introduction

The course and outcome of cutaneous leishmaniasis caused by the parasite *Leishmania major* depends on the type of immune response mounted by the host, whereby the humoral and the cell-mediated responses are, respectively, associated with the susceptibility and resistance to the prevailing infection (Cunningham, 2002), because these parasites have the capacity to escape the humoral response by residing in the phagolysosomes of macrophages. The type of the immune response itself depends on many factors including the genetic background of the host (Sakthianandeswaren et al., 2009), the cytokine milieu (Liu and Uzonna, 2012), and the dose of the injected parasite. Regardless of the course and outcome of the infection, the induced inflammatory response involves hyperalgesia, which is the enhancement of pain sensitivity to noxious stimuli and spontaneous pain (Driessen, 2007; Cervero, 2009) due to sensitization of nociceptors (hypernociception). Sensitization of nociceptors is of two types; peripheral sensitization and central sensitization. Peripheral sensitization is an increased responsiveness to stimuli by the peripheral ends of nociceptors. These neurons transfer signals from peripheral targets (skin, joints, muscle, and viscera) to the central nervous system (Spinal cord and brainstem) (Woolf and Ma, 2007). Even though pain hypersensitivity is mainly based on peripheral sensitization, high levels of activity lead to the activation of dorsal horn nociceptors resulting in the modification of sub threshold innocuous stimuli so that they activate second order neurons in the dorsal horn giving rise to a central sensation of pain which is so called allodynia (Kidd and Urban, 2001).

**FIGURE 1:**
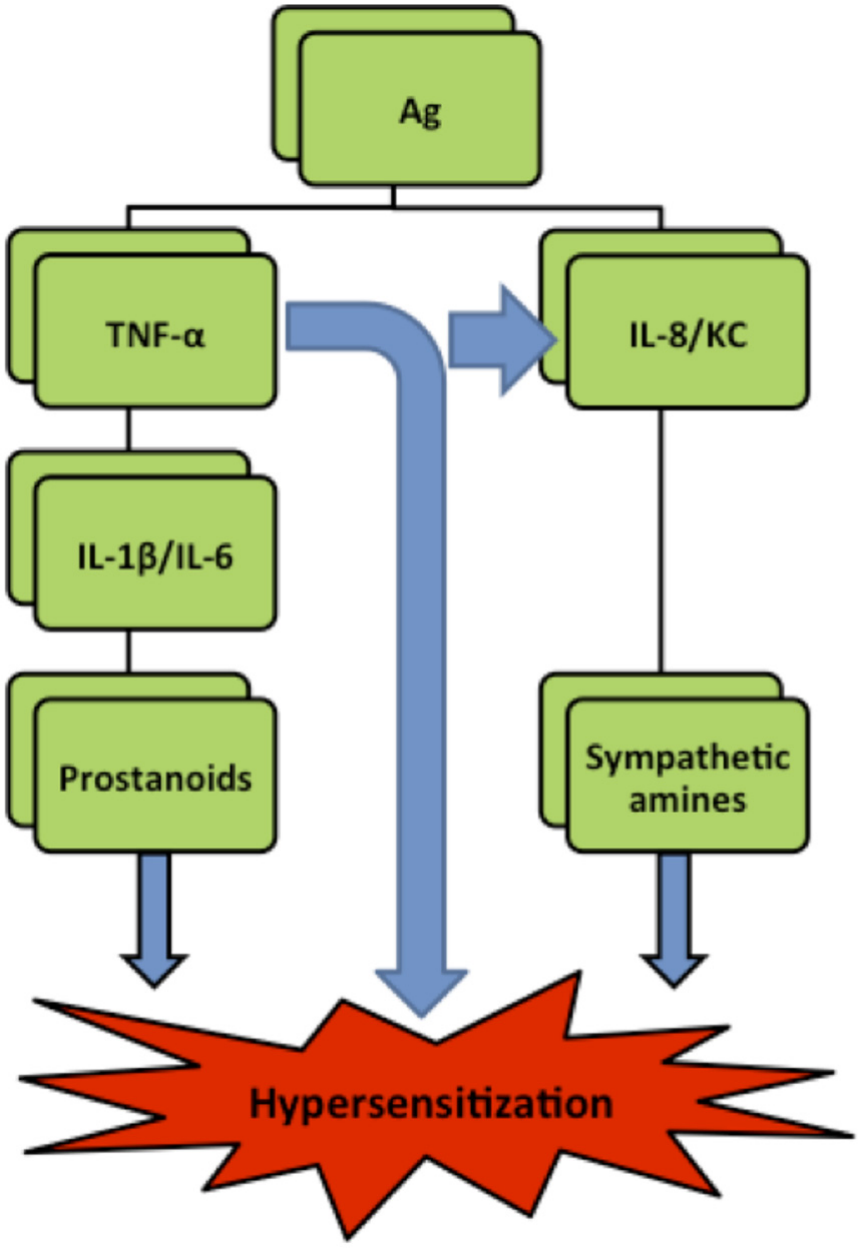
**Simplified hyperalgesia pathways in rats.** Ag (antigen), IL (interleukin), TNF-**α** (Tumor necrosis factor-**α**), KC (Keratinocyte derived chemokine) (Verri et al., 2006).

Hypernociception is induced by the direct action of the final inflammatory mediators—prostaglandins and sympathetic amines—on peripheral nociceptors. Subsequently, the secondary signaling pathways [mediated by cyclic AMP (cAMP), protein kinase A (PKA), and protein kinase C (PKC)] are triggered lowering the nociceptor threshold and increasing neuronal membrane excitability (Amaral et al., 2008). Although prostaglandins and sympathetic amines are suspected to be the final sensitizers of the nociceptors (Safieh-Garabedian et al., 2002), their production is preceded by the release of a cascade of cytokines and chemokines (Cunha et al., 2005). It is widely accepted that tumor factor α (TNF-α), which is a potent pro-inflammatory cytokine and is rapidly produced in large quantities by macrophages in response to inflammatory stimuli (Verri et al., 2006), can initiate two independent and parallel pathways. One cascade involves the release of interleukin 1β (IL-1β) and IL-6 leading to prostanoids production while the other cascade involves interleukin 8 (IL-8) (or Keratinocyte-Derived Chemokine/KC in mice), leading to production of sympathomimetic mediators (Cunha et al., 1992; **Figure 1**).

As to cutaneous leishmaniasis, a persistent hyperalgesia was reported in BALB/c mice infected with high dose (Kanaan et al., 2000) and a short lived one in those infected with low dose of the parasite (Karam et al., 2006). While assessing the effect of thymulin (Kanaan et al., 2000), IL-10 (Karam et al., 2007), and IL-13 (Karam et al., 2011) on *L. major* induced hyperalgesia, it was shown that those substances could reverse the low pain thresholds during the treatment period. This effect was accompanied by the reduction of the increased levels of nerve growth factor (NGF) and IL-1β and by a further upregulation of IL-6 (Karam et al., 2006). However, although hyperalgesia was restored after stopping the treatment with IL-10 and IL-13, the levels of IL-6 remained upregulated (Karam et al., 2007) and interestingly those of IL-1β (in the case of IL-13 treatment) remained down-regulated (Karam et al., 2011) at most time points of the whole experimental period. By correlating the pain thresholds with the cytokines levels time courses as well as the treatment period, it was concluded that IL-1β and IL-6 do not play a direct role and that other mediators are involved in *L. major* induced hyperalgesia at least during later stages of the infection. Taken together, these results suggest that a more pronounced role can be attributed to the sympathetic amines as the final mediators of *L. major* induced hyperalgesia; therefore TNF-α and/or (KC) should most probably be the intermediate mediators. Consequently, investigating the role of cyclooxygenase inhibitors (as indomethacin) as well as beta blockers (as atenolol) should help in having a better idea about the pathway leading to *L. major* induced hyperalgesia in mice. Indomethacin is known to inhibit the cyclooxygenases 1 and 2 (COX-1/COX2) responsible for the production of PGE_2_ resulting in the reduction of hyperalgesia. The inhibition of hyperalgesia is mediated through the inhibition of the pro-inflammatory cytokine IL- 1β (Cunha et al., 1991).

Atenolol is known to reduce hyperalgesia to bradykinin, LPS (Ferreira et al., 1993), kallidin, and DABK (des Arginine Bradykinin) (Poole et al., 1999). This reduction of hyperalgesia was suggested to be through direct effect on the levels of IL-8 (Cunha et al., 1991). Therefore, the aim of this study was to investigate the effect of daily treatment with Indomethacin and the β-blockers atenolol on *L. major*-induced inflammation in mice with respect to hyperalgesia and levels of IL-lβ, TNF-α and KC.

## Materials and Methods

### Experimental Animals

Adult female BALB/c mice, provided by the animal house at the University of Balamand (20–30 g) were used in all the experiments performed in this study. The animals were housed under optimal conditions of light and temperature (12 h light and 12 h dark cycles; 22 ± 3°C) and received solid food and water *ad libitum*. During the period of the experiments, the mice were kept in groups of five or six, in clear plastic cages with solid floors covered with 3–6 cm of saw dust. All experimental procedures were carried out with ethics committee approval from the University of Balamand and with strict adherence to the ethical guidelines for the study of experimental pain in conscious animals (Zimmerman, 1983).

### Parasite Culture and Preparations

*Leishmania major* (MHOM/SU/73/5 ASKH) parasites (purchased from the London School of Hygiene and Tropical Medicine, London, UK) were grown at 22 ± 1°C to 25 ± 1°C in a standard monophasic medium and sub-cultured weekly. The medium was made of nutrient broth (Peptic digest of animal tissue 5 g/L, Sodium chloride 5 g/L, Beef extract 1.500,Yeast extract 1.5 gms/L and final pH of 7.4 ± 0.2 at 25°C) supplemented with 20% fetal bovine serum, 100 IU/ml of penicillin, and 100 IU/ml of streptomycin (Sigma). The medium containing the promastigote forms of the parasite were centrifuged for 15 min at 2500 rpm and the parasites were resuspended in 1 ml nutrient broth medium. The parasite count was determined in a Trypan blue solution using a hemocytometer and readjusted to have 4 × 10^6^ parasites per 50 μl nutrient broth medium for a high dose of *L. major*. All mice received intraplantar injection subcutaneously (s.c.) with the parasite in their left hind paw.

The reported results are based on two sets of experiments each involving different groups of mice (*n* = 5 in each) which were used either for the behavioral pain tests or the determination of the levels of cytokines in the paws of the mice.

### Behavioral Measurements

#### Experimental Groups

Seven groups of mice were subjected to different pain tests 3 days before any treatment, and then five groups received i.pl. injection high dose of *L. major* (4 × 10^6^ promastigotes/50 μl nutrient broth medium/left hind paw): one group was left with no treatment, another one was treated with indomethacin (5 mg/kg) and three groups were treated with atenolol (0.5, 1.0, 2.0 mg/kg/100 μl, respectively). All treated groups received daily i.p. injections for 7 days. The last two groups consisted of naïve (neither infected nor treated) mice and those injected s.c. in the left paw with the culture medium and both were used also as control (**Table 1**).

**Table 1:** Experimental groups used in the behavioral tests.

Group	Parasitic injections (s.c 4 × 10^6^ parasites)	Treatment (i.p.)
1	None	None
2	None	Parasites culture medium
3	*L. major*	None
4	*L. major*	5.0 mg/kg Indomethacin
5	*L. major*	0.5 mg/kg Atenolol
6	*L. major*	1.0 mg/kg Atenolol
7	*L. major*	2.0 mg/kg Atenolol


All groups were subjected to pain tests daily except on weekends following the injections for a period of 24 days.

#### Pain Tests

The hot plate (HP) and the tail flick (TF) tests were used to assess the thermal pain thresholds.

##### The tail immersion test

The tails of the mice were immersed into a water bath of distilled water, the temperature of which being adjusted at *T* = 50°C (±0.5°C). The time interval between the immersion of the tail and the tail flicking reaction was recorded using a digital stopwatch with a 1/100 second precision. Each animal was subjected to this test three times with a three minutes interval between consecutive measurements and the average was recorded (Kanaan et al., 1996).

##### The hot plate test

Each animal was placed on a hotplate analgesia meter (Harvard Apparatus) with the temperature being adjusted at *T* = 51°C (±0.5°C). The latency of paw licking or jumping or urinating was taken.

### Immunoassays

#### Tissue Processing Assay

Four sets of mice, each containing five groups (*n* = 5), were used in this part of the study. Two sets were infected s.c. with *L. major* (4 × 10^6^ promastigotes/50 μl nutrient broth medium/left hind paw) and one of them was treated intraperitoneally (i.p.) with atenolol (2.0 mg/kg) for 7 days. The other two sets were used as control (one consists of naive mice and the other of mice infected with *L. major*-free broth medium).

The skin of both right (non-infected) and left (infected) hind paws of mice of the different sets was removed under deep anesthesia with chloroform at days 2, 4, 6, 13, 21 post-infection.

The paws were individually homogenized (using IKA T 10 basic ULTRA-TURRAX homogenizer) for 1 min at a speed of 20,000 r.p.m. in 1.2 ml of homogenization buffer. The homogenization buffer was prepared by dissolving 139.83 mM NaCl. 2.68 mM KCl, 1.47 mM KH_2_PO_4,_ 8.09 mM Na_2_HPO_4_ in 100 ml double distilled water [(pH 7.2–7.4). It constituted of 399.58 mM of NaCl, 0.5 g bovine serum albumin (BSA), 50 μl of tween 20 and two tablets protease inhibitor to 100 ml of PBS. The homogenized samples were centrifuged at 13000 *g* for 60 min at 4°C; supernatants were removed and stored in pyrogen/endotoxin-free tubes at –82°C until further usage.

The supernatants were than assayed for levels of IL-1β, TNF-α, and KC using a two-site enzyme-linked immunosorbent assay (ELISA) kits (QIAGEN). For all assays, microtiter plates (Corning) were setup according to the manufacturer’s instructions and read at 450 nm using Ascent software (Epoch-Biotek).

### Paw Thickness

In order to make sure that the strain of *L. major* causes cutaneous leishmaniasis, the paw thickness of one group of mice that received i.pl. injection with high dose of the parasite (4 × 10^6^ promastigotes/50 μl nutrient broth medium/left hind paw) was measured using a digital caliper on a weekly basis for 32 weeks post-infection.

### Data Analysis

Values of pain tests represent the average of three trials at each time interval and plotted as time latency versus duration of test. For the immunoassays, the measured levels of each cytokine were averaged on every tissue removal date for each experimental group. Comparison among groups was located using a one-way ANOVA followed by a Bonferoni *post hoc* test. Results having *p* < 0.05, *p* < 0.01, and *p* < 0.001 were considered significant.

## Results

### *L. major* Infection

#### Effect of High Dose *L. major* on the Paw Thickness of Mice and Pain Thresholds

In order to demonstrate the *L. major* infection in mice, the paw thickness of the infected paws with high dose of *L. major* (4 × 10^6^ promastigotes/50 μl nutrient broth medium/left hind paw) was assessed using a digital caliper.

**FIGURE 2:**
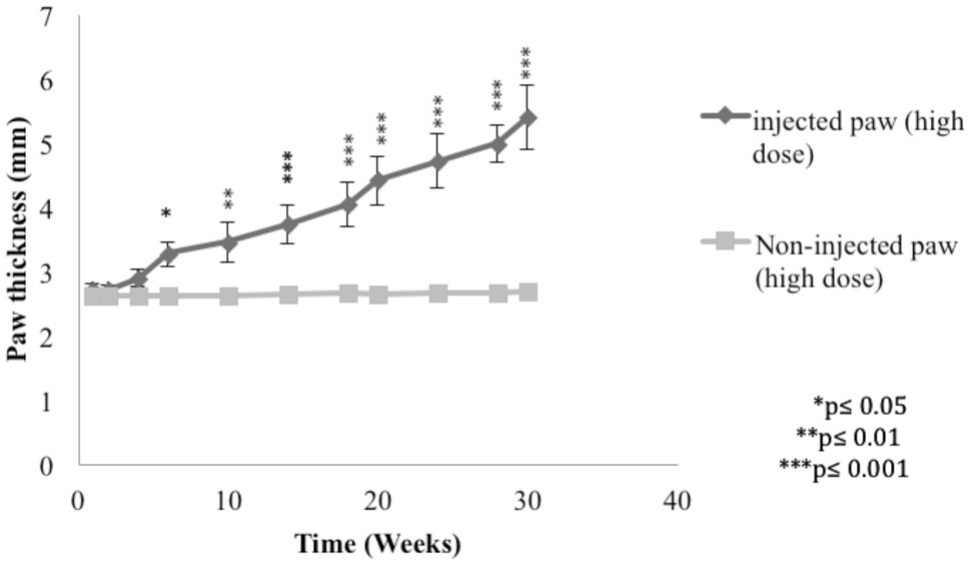
**Effect of *Leishmania major* infection on the paw thickness in mice infected with high dose (4 × 10^6^ promastigotes/50 μl nutrient broth medium/left hind paw).** Each result is the mean of 5 mice ± SEM and the degree of significance of the difference was calculated with reference to the non-infected paw.

The subcutaneous injection of a high dose of *L. major* caused a significant change in the paw thickness of the infected paw of infected mice starting at week 6 post-infection and continued to increase throughout the period of the experiment reaching 5.39 ± 0.49 mm at week 30 post-infection for the infected paw as compared to 2.69 ± 0.12 mm for the non-infected paw (*p* < 0.001; **Figure 2**).

**FIGURE 3:**
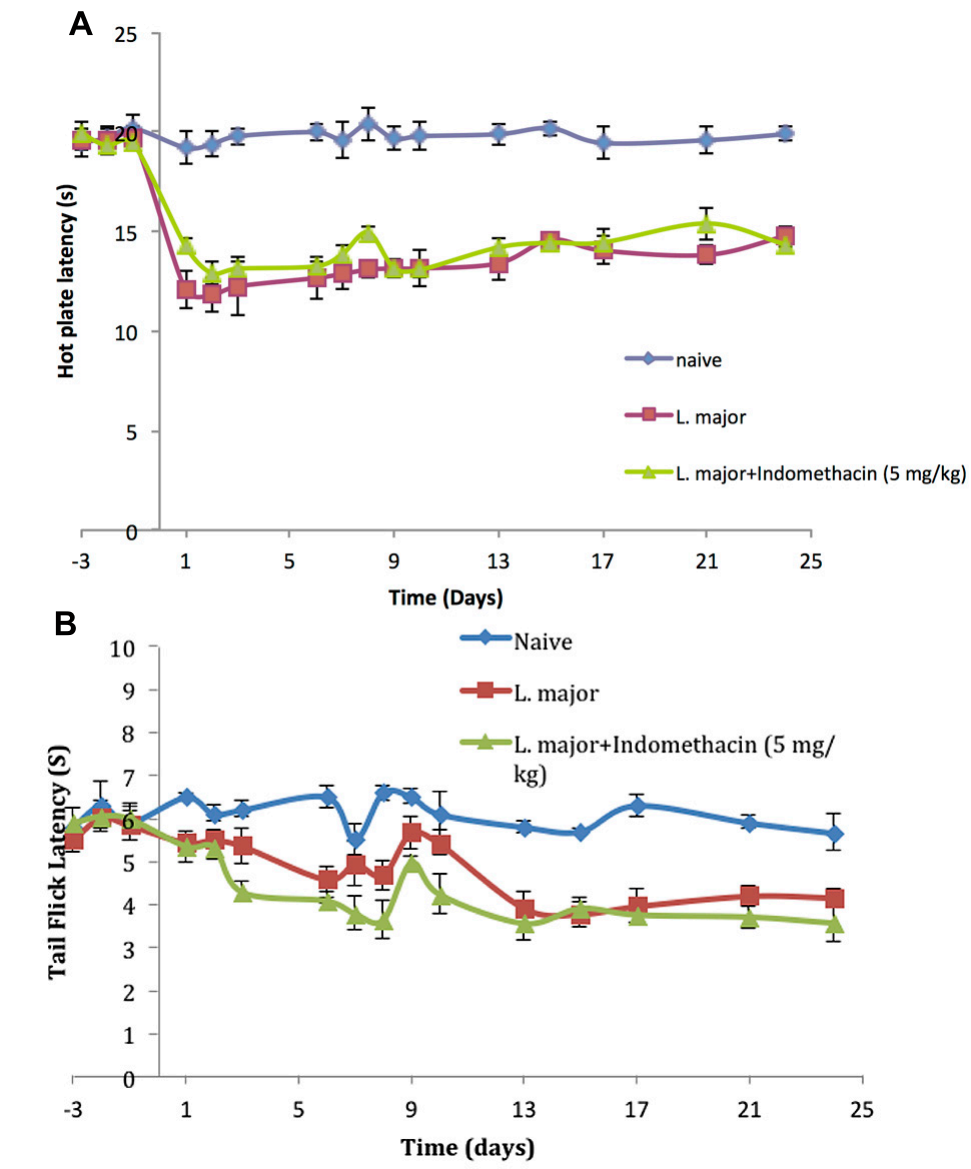
**The time course of the effect of the daily intraperitoneal (i.p.) injection of indomethacin on *L. major* (high dose) induced hyperalgesia: as assessed by **(A)** the Hot Plate (HP) Test and **(B)** the Tail Flick (TF) Test.** Mice were injected with 4 × 10^6^ promastigotes per 50 μl per left hind paw and another group also with 5.0 mg/kg of indomethacin for 7 consecutive days. Each result is the mean of 5 mice ± SEM and the degree of significance was calculated with reference to indomethacin non-treated mice.

The injection of *L. major* produced a significant decrease in the HP latency (as calculated with reference to non-infected mice). It rapidly dropped starting day 2 post-infection from 19.4 ± 0.61 s in non-infected mice to 11.8 ± 0.83 s in the infected mice (*p* < 0.001; **Figure 3A**).

As to the TF latency, the infection with high dose of *L. major* caused a significant decrease at day 6 post-infection. It dropped from 6.5 ± 0.25 s to 4.58 ± 0.28 s (*p* < 0.001). TF latency showed another significance decrease at day 13 post infection reaching 3.92 ± 0.36s (*p* < 0.01) as compared to non-infected mice 5.8 ± 0.134 s and this effect was sustained through the whole experimental period (**Figure 3B**).

### Treatment with Indomethacin

#### Pain Thresholds

The infection of mice with high doe of *L. major* resulted in a significant decrease in the pain thresholds as assessed by the HP and TF tests. However, treatment with daily injections of indomethacin (5.0 mg/kg) for 7 consecutive days did not result in any significant difference in the HP latencies of the mice infected with high dose *L. major* and treated compared to those infected with *L. major* and not treated with indomethacin at any of the time points (all *p* values > 0.05; **Figure 3A**).

In addition, daily treatment with indomethacin did not affect the TF latencies as compared to the indomethacin non-treated group at any time point during the experimental period (*p* > 0.05; **Figure 3B**).

### Treatment with Atenolol

#### Pain Thresholds

**FIGURE 4:**
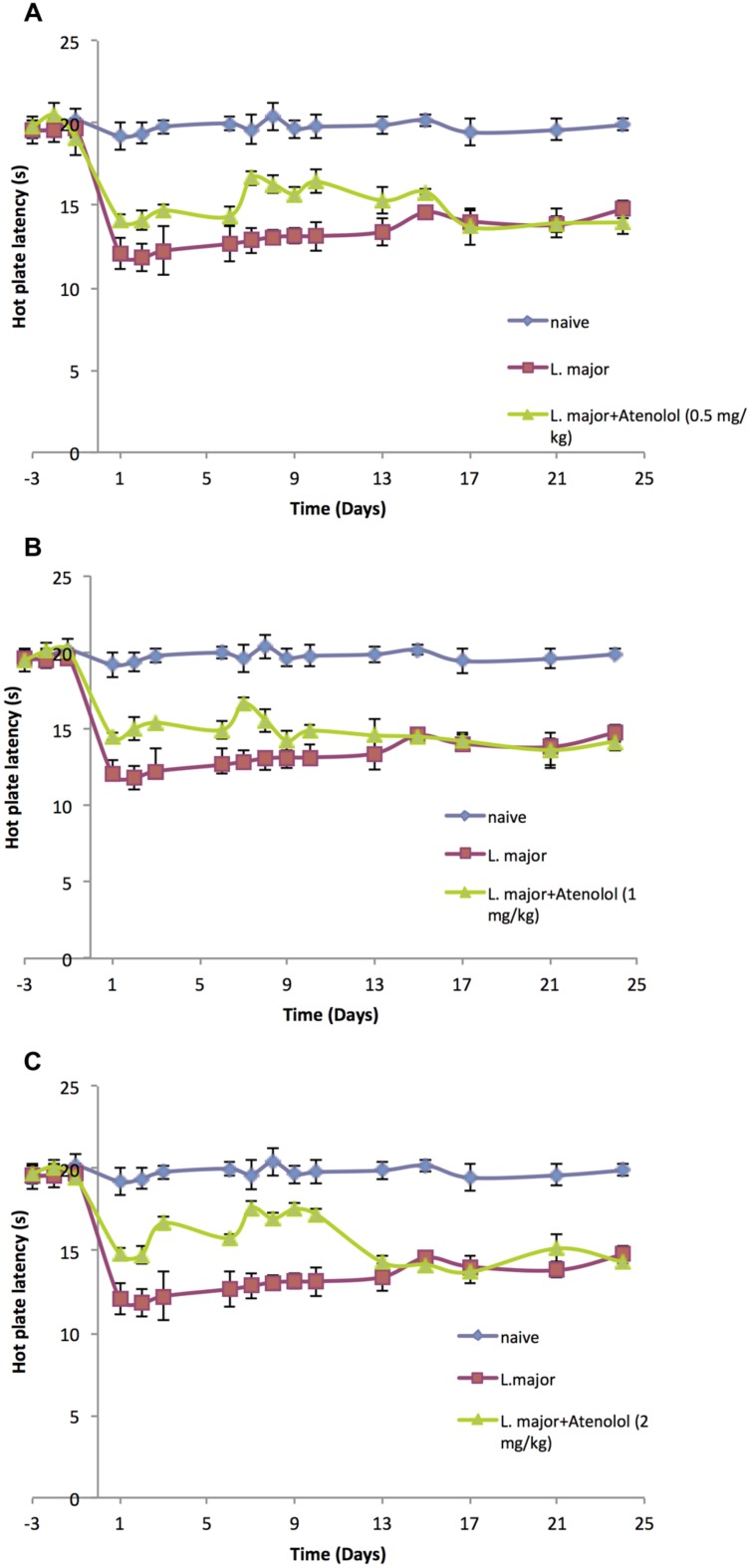
**The time course of the effect of the daily i.p. injection of atenolol on *L. major* (high dose) induced hyperalgesia as assessed by the HP Test.** Mice were injected with 4 × 10^6^ promastigotes per 50 μl per left hind paw and three groups were also treated with Atenolol 0.5 mg/kg **(A)**, or 1.0 mg/kg **(B)**, or 2.0 mg/kg **(C)** for 7 consecutive days. Each result is the mean of 5 mice ± SEM and the degree of significance was calculated with reference to atenolol non-treated mice.

Treatment with atenolol (0.5, 1.0, 2.0 mg/kg) for 7 consecutive days produced a significant reduction in the high dose *L. major*-induced hyperalgesia during the period of treatment. The HP latency increased at day 1 post-infection from 11.8 ± 0.83 s in the mice infected with *L. major* alone to 14.1 ± 0.41 s in the group treated with 0.5 mg/kg atenolol (*p* < 0.01; **Figure 4A**). This significant difference in HP latency was also observed in the group treated with 1.0 mg/kg atenolol (14.5 ± 0.22 s; **Figure 4B**), and the group treated with 2.0 mg/kg atenolol (14.8 ± 0.36 s; **Figure 4C**). However, the difference between the HP latencies of the treated mice and the infected mice became significant especially from day 7 through day 10 when the maximum effect was reached. Thereafter, the levels started to decrease to reach a minimum of 13.97 ± 0.75 s (0.5 mg/kg/100 μl), 14.18 ± 0.59 s (1.0 mg/kg/100 μl), and 14.32 ± 0.83 s (2.0 mg/kg/100 μl) at day 24 post-infection as compared to 14.76 ± 0.53 s in the mice infected with high dose of *L. major* (**Figures 4A–C**, respectively) and the difference between those two groups became insignificant.

**FIGURE 5:**
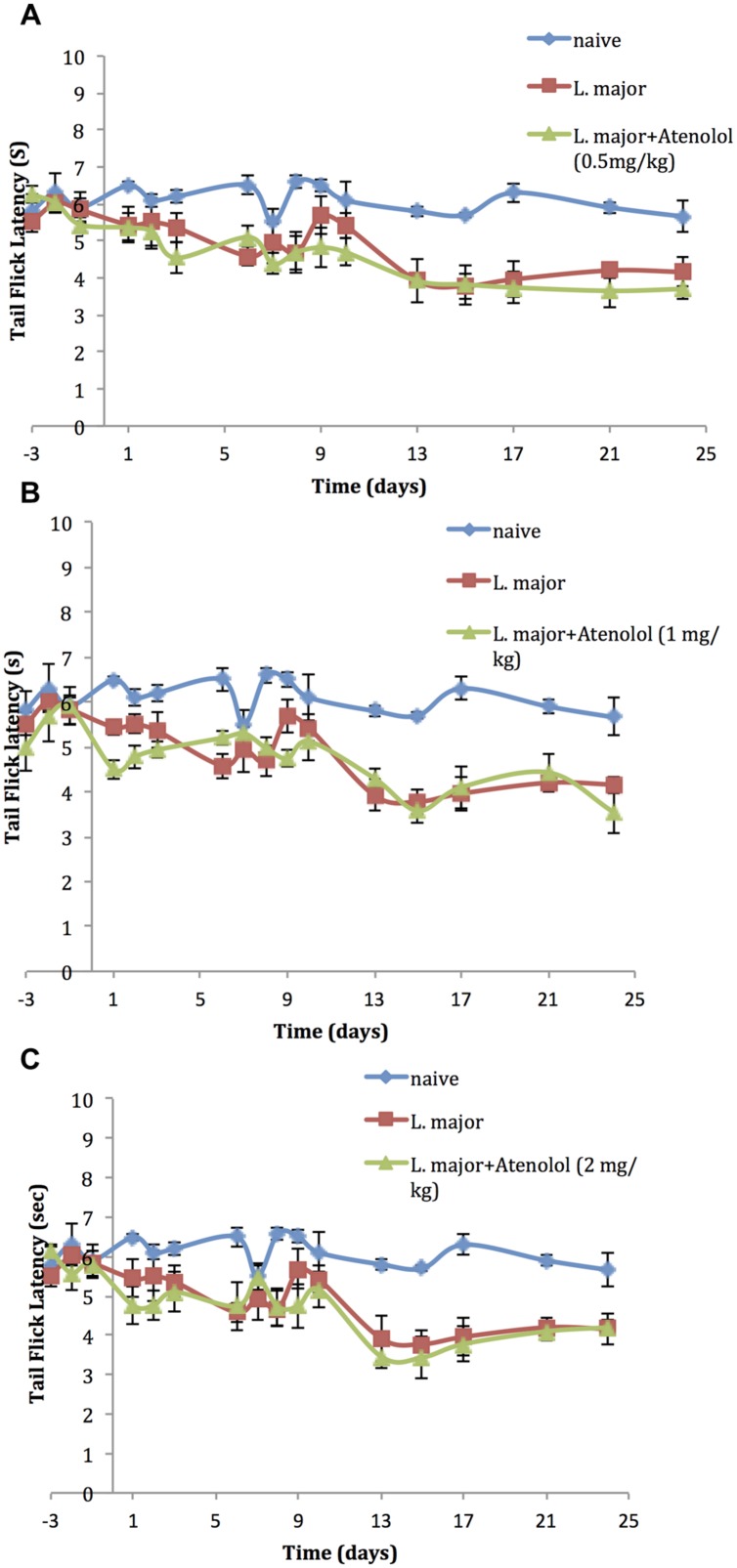
**The time course of the effect of the daily i.p. injection of Atenolol on *L. major* (high dose) induced hyperalgesia as assessed by the TF test.** Mice were injected with 4 × 10^6^ promastigotes per 50 μl per left hind paw and three groups were also treated with Atenolol 0.5 mg/kg **(A)**, or 1.0 mg/kg **(B)**, or 2.0 mg/kg **(C)** for 7 consecutive days. Each result is the mean of 5 mice ± SEM and the degree of significance was calculated with reference to atenolol non-treated mice.

On the other hand, daily treatment with i.p. atenolol did not affect the TF latency as compared to the non-treated group at any time spot during the experimental period (*p* > 0.05; **Figures 5A–C**).

Although the three concentrations of atenolol were efficient in reducing the *L. major* induced hyperalgesia, the highest concentration (2.0/mg/kg) seems to be the most effective one at most post infection dates. Therefore, this concentration was used to perform the ELISA assays.

#### Cytokines

##### Effect of atenolol (2.0 mg/kg) on the level of TNF-α in mice infected with high dose of *L. major*

**FIGURE 6:**
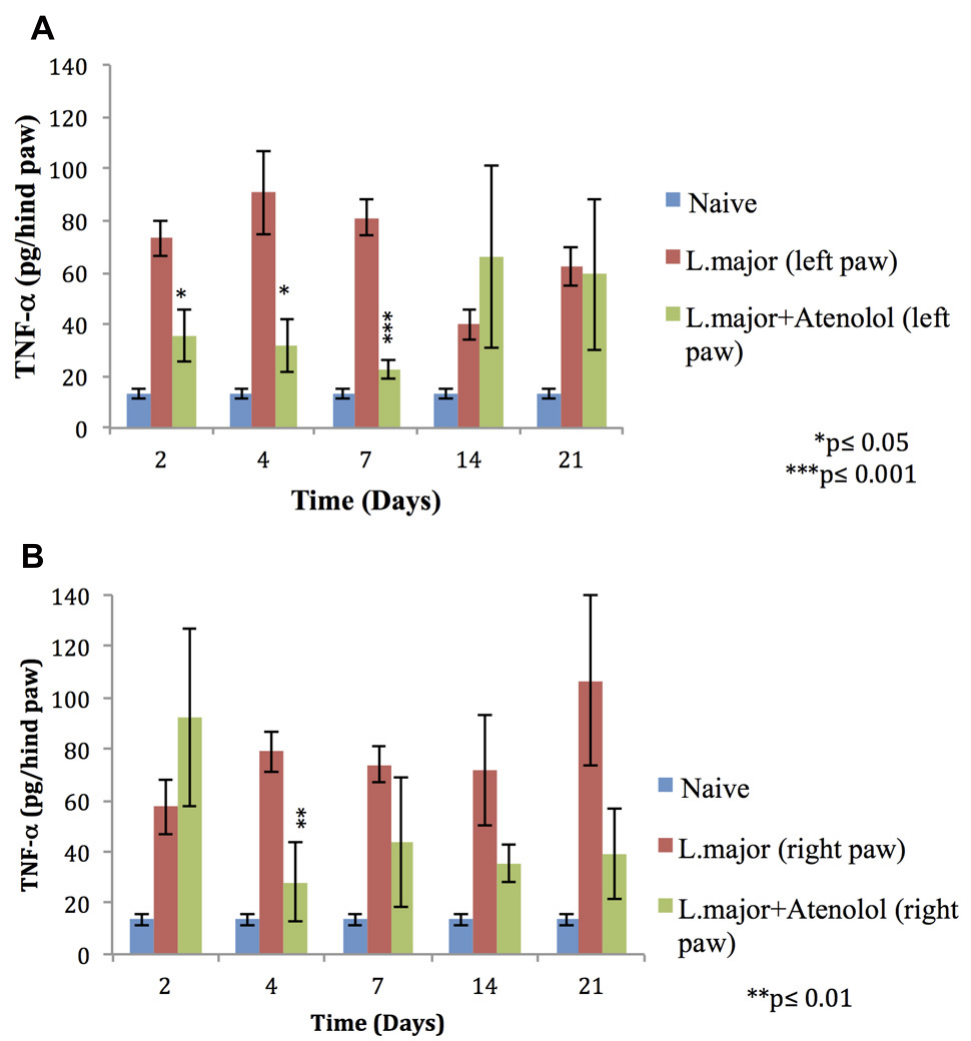
**Effect of the daily i.p. injection of atenolol on the level of TNF-α in the paws of mice infected with high dose (4 × 10^6^ promastigotes/50 μl nutrient broth medium/left hind paw) of *L. major*: **(A)** left (infected) paws **(B)** right (non-infected) paws.** Mice were injected with 2.0 mg/kg of atenolol (i.p.) for 7 consecutive days and the levels of TNF-α were assessed at days 2, 4, 7, 14, and 21 post-infection. Each result is the mean of 5 mice ± SEM and the degree of significance was calculated with reference to atenolol non-treated infected mice.

The subcutaneous injection of a high dose of *L. major* (4 × 10^6^ promastigotes/50 μl nutrient broth medium/left hind paw) caused a significant increase in the levels of TNF-α, in the infected (left) and non-infected (right) hind paws at most time points of the experiment (till day 21 post-infection) (**Figures 6A,B**).

Atenolol reduced significantly the levels of TNF-α in the infected paws of mice starting day 2 post-infection becoming more significant at day 7 post-infection when the levels of TNF-α reached 22.79 ± 3.77 pg/hind paw in the paws of treated mice as compared to 80.98 ± 6.83 pg/hind paw in the infected paws of non-treated mice (*p* < 0.001; **Figure 6A**).

As for the right (non-infected) paws of mice infected with high dose *L. major* the effect of atenolol on the levels of TNF-α was only significant at day 4 post infection becoming 28.29 ± 15.27 pg/hind paw as compared to 79.01 ± 7.91 pg/hind paw for the infected atenolol untreated mice (*p* < 0.01; **Figure 6B**).

##### Effect of Atenolol 2.0 mg/kg on the Level of IL-1 in mice infected with high dose of *L. major*

**FIGURE 7:**
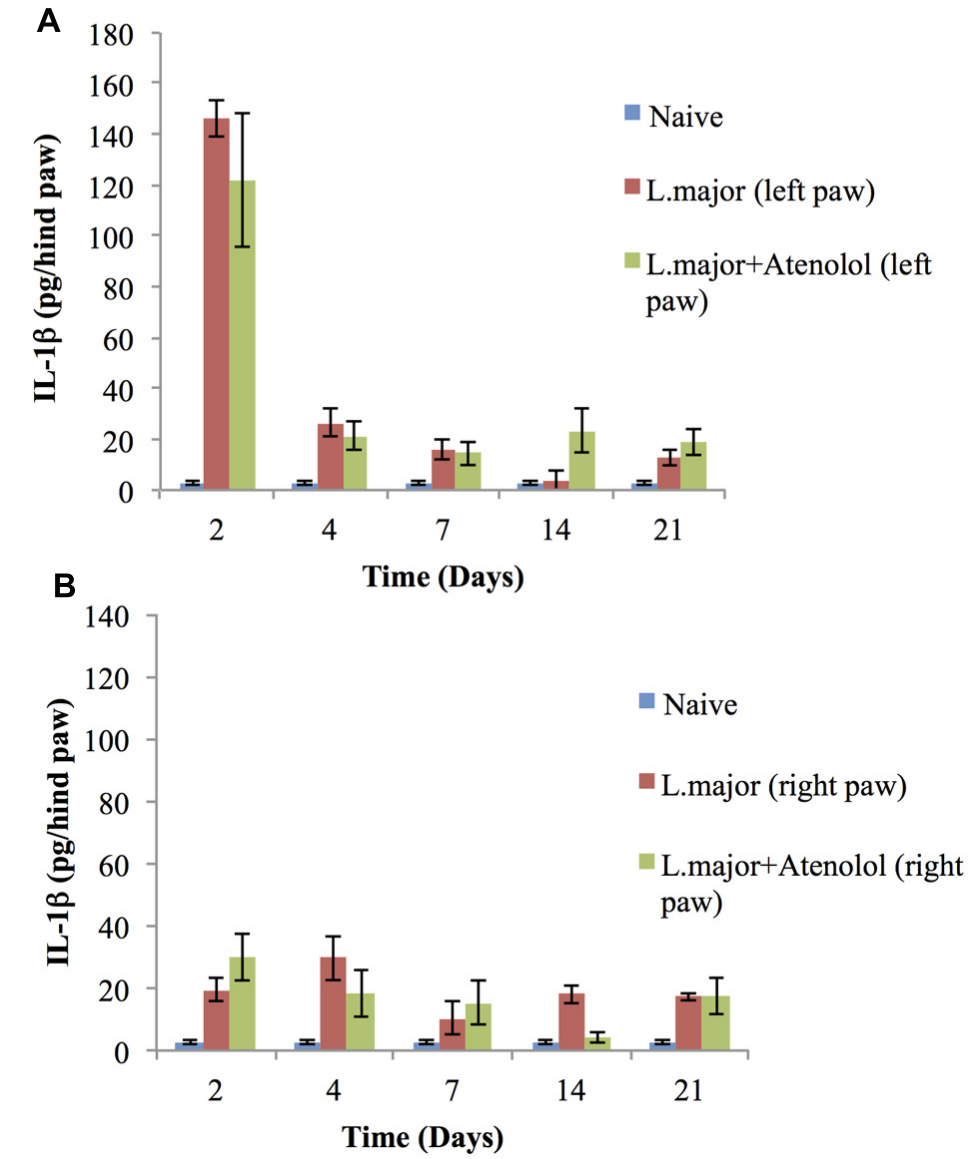
**Effect of the daily i.p. injection of atenolol on the level of IL-lβ in the paws of mice infected with high dose (4 × 10^6^ promastigotes/50 μl nutrient broth medium/left hind paw) of *L. major*: **(A)** left (infected) paws **(B)** right (non-infected) paws.** Mice were injected with 2.0 mg/kg of atenolol (i.p.) for 7 consecutive days (and the levels of IL-lβ were assessed at days 2, 4, 7, 14, and 21 post-infection. Each result is the mean of 5 mice ± SEM and the degree of significance was calculated with reference to atenolol non-treated mice.

The subcutaneous injection of a high dose of *L. major* (4 × 10^6^ promastigotes/50 μl nutrient broth medium/left hind paw) caused a significant change in the levels of IL-1 in the infected paws of mice starting day 2 post-infection when it increased from 2.75 ± 0.67 pg/hind paw in the non-infected mice to 146 ± 7.32 pg/hind paw (*p* < 0.001). This significant increase was sustained till day 7 post-infection (**Figure 7A**). On the other hand, the levels of IL-1β in the non-infected paw (right) showed no significant change at any time point when compared to the control mice (**Figure 7B**).

Daily treatment with atenolol (2.0 mg/kg) for 7 days had no significant effect, at any date post-infection, on the level of IL-1β neither in the infected paws nor in the non-infected paws of mice infected with *L. major* as compared to those infected with *L. major but* not treated with atenolol (all *p* values > 0.05) (**Figures 7B**).

##### Effect of Atenolol (2.0 mg/kg) on the level of KC in mice infected with high dose of *L. major*

**FIGURE 8:**
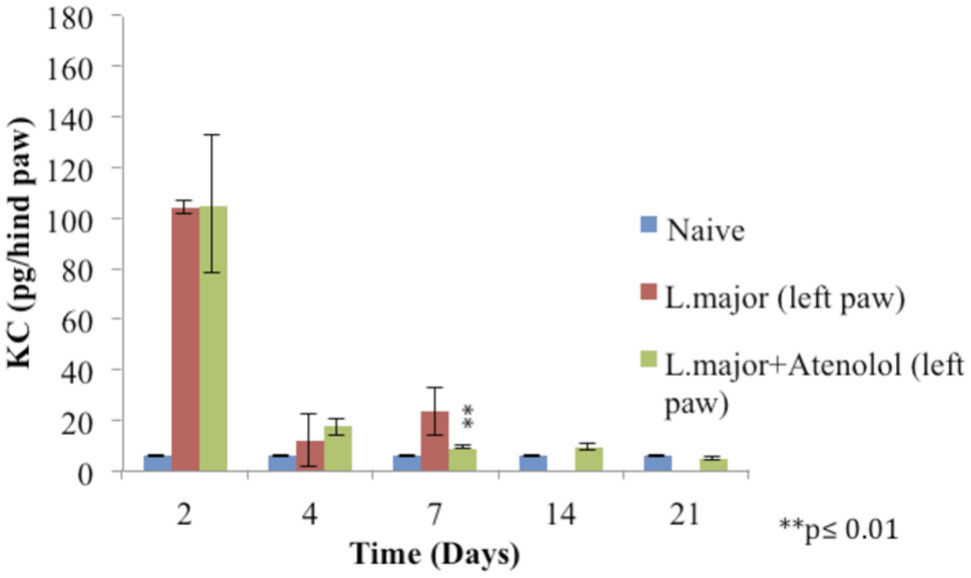
**Effect of the daily i.p. injection of atenolol on the level of KC in the paws of mice infected with high dose (4 × 10^6^promastigotes/50 μl nutrient broth medium/left hind paw) of *L. major*: paws removed from the left paws of mice.** Mice were injected with 2.0 mg/kg of atenolol (i.p.) for 7 consecutive days and the levels of KC were assessed at days 2, 4, 7, 14, and 21 post-infection (at day 14 and 21 the level of KC in the group infected is not detected). Each result is the mean of 5 mice ± SEM and the degree of significance was calculated with reference to atenolol non-treated mice.

The subcutaneous injection of a high dose of *L. major* (4 × 10^6^ promastigotes/50 μl nutrient broth medium/left hind paw) caused a significant increase in the level of KC at day 2 post-infection in the infected hind paw of the mice (104 ± 2.28 pg/hind paw) (*p* < 0.001) as compared to the level in the control group (5.83 ± 0.124 pg/hind paw). A significant increase was also shown at day 7 when the KC levels became 23.33 ± 9.18 in the infected paws of infected mice. The levels of KC at day 14 and 21 were not detectable (**Figure 8**).

Treatment with daily injections of atenolol (2.0 mg/kg) for 7 days resulted in no significant difference at day 2 and 4 post infection between the levels of KC in the paws of mice infected with *L. major* and those infected with the parasite and treated with atenolol (*p* > 0.05). The level of KC at day 7 showed a decrease in the paws of mice infected and treated (8.948 ± 0.63 pg/hind paw) as compared to the infected and non-treated mice (23.33 ± 9.18 pg/hind paw), the *p*-value was <0.01 (**Figure 8**).

As for the level of KC in the right (non-infected) paws, it was not detected at all time points of the experiment.

## Discussion

It is generally well accepted that the inflammatory hypernociception, in many animal models, depends on two hyperalgesic pathways involving the sympathetic amines and/or prostanoids as final mediators. In the cutaneous leishmaniasis model, the subcutaneous injection of high dose of *L. major* in the paws of BALB/c mice causes a sustained hyperalgesia accompanied by an increase in the levels of pro-inflammatory cytokines IL-lβ and NGF (Kanaan et al., 2000) while infection with a low dose of the parasite causes a short lived hyperalgesia accompanied by the upregulation of IL-1β and IL-6 (Karam et al., 2006).

However, while assessing the effect of IL-10 and IL-13 on *L. major* induced hyperalgesia in BALB/c mice, it has been shown that IL-lβ and IL-6 do not play a direct role in evoking or sustaining the low pain thresholds (Karam et al., 2011) suggesting that this hyperalgesia is mainly mediated by the pathway leading to the production of sympathetic amines.

Therefore, we started by re-evaluating the effect of *L. major* infection on the pain thresholds of BALB/c mice. In line with previous studies (Kanaan et al., 2000; Karam et al., 2007), we demonstrated that the subcutaneous injection of a high dose of *L. major* causes a sustained hyperalgesia as measured by the significant decrease in the pain thresholds toward the HP test. In addition to peripheral sensitization, we have shown that *L. major* infection results in decreased latency in TF test starting day 12 post-infection, suggesting a central sensitization phenomenon (Mahesh et al., 2010).

In order to confirm or reject any direct role of the prostanoid pathway in the *L. major* induced hyperalgesia, mice were treated with indomethacin, which is a cyclooxygenase inhibitor. Our results showed that indomethacin caused no significant effect on *L. major* induced low pain thresholds providing a further proof that this hyperalgesia model is mainly dependent on the sympathetic amines pathway.

Accordingly, we assessed the effect of atenolol, a β-blocker and β1-adrenoceptor antagonist on the *L. major* induced pain thresholds; Atenolol is known to limit hyperalgesia in several models other than leishmaniasis through blocking the sympathetic amines. The i.p. injection of atenolol induced a dose-dependent (0.5, 1.0, 2.0 mg/kg) reduction in the peripheral hyperalgesia, as assessed by the HP test, during the period of treatment. However, atenolol did not have any significant effects on the central sensitization as assessed by the TF test.

The above results were encouraging to investigate the effect of atenolol on the levels of some key cytokines that might be involved in the process of hyperalgesia. Therefore, we assessed the levels of TNF-α, IL-lβ and KC in the paws of *L. major* infected mice treated with 60 mg/100 μl/mouse for 7 consecutive days.

There have been correlations between tissue levels of TNF-α and pain and hyperalgesia in a number of painful diseases (Barnes et al., 1992; Shafer et al., 1994; Tak et al., 1997; Lindenlaub and Sommer, 2003). In addition there is accumulating evidence that TNF-α, which is released by activated glial cells, is an important mediator of pain and hyperalgesia in the central nervous system (Watkins et al., 2007). Upon *L. major* infection, no significant change has been detected in the levels of TNF-α in infected BALB/c mice (Kanaan et al., 2000). In contrast to previous studies, our results showed that following infection with *L. major* and using the same mouse strain, the levels of TNF-α increased at all time points of the experiment at both the local infected site and the distal non-infected site, suggesting a major role of TNF-α in the observed peripheral and central sensitization.

Our results showed that atenolol is able to reduce the levels of TNF-α in the infected paws of the mice throughout the treatment period. Thereafter, the levels of TNF-α started to increase reaching values very close to levels of this cytokine in the paws of mice infected with high dose of *L. major* but not treated with atenolol. This time course of the levels of TNF-α correlates very well with that of the observed peripheral hyperalgesia as assessed by the HP test. As to the levels in the non-infected paws, atenolol seems to have a reducing effect trend on the TNF-α levels starting day 4 post-infection and throughout the whole experimental period (even when the treatment was stopped); however, atenolol treatment did not reduce the TF low pain thresholds suggesting that this beta blocker can, most probably by down-regulating the TNF-α levels, reduce the peripheral but not the central hyperalgesia.

Although several studies reported that *L. major* induced hyperalgesia is mediated through cytokines, in particular a sustained up-regulation of IL-lβ (Saade et al., 2000; Karam et al., 2007), Karam et al. (2011) have shown that IL-lβ has no direct role in *L. major* induced hyperalgesia using the same dose of parasites and same mouse strain. Our results are in agreement with the latter study since *L. major* infection caused a sustained peripheral hyperalgesia while the levels of IL-lβ were significantly increased just during the first few days of infection. Moreover, and as expected, atenolol treatment did not have any significant effect on the levels of IL-lβ which remained up-regulated while the peripheral hyperalgesia was reduced during the period of treatment. These results suggest that mediators other than IL-lβ are involved in *L. major* induced peripheral hyperalgesia.

From the other side, the tendency of the levels of IL-lβ to increase in the non-infected paws of the mice due to *L. major* infection, and the inability of atenolol to significantly reduce them parallel to the low TF threshold values, might suggest a possible role of this pro-inflammatory cytokine in the observed central sensitization. However, since indomethacin did not reverse those low TF threshold values, and taking into consideration that hyperalgesia evoked by IL-lβ was not attenuated by atenolol (Cunha et al., 1991), and that, in mice, IL-lβ produces hypernociception through the induction of PGE2 (Cunha et al., 2005), the latter hypothesis is not favored except if IL-lβ is mediating hyperalgesia via mediators other than prostanoids.

Therefore, more studies are required to investigate the serial levels of pro-inflammatory cytokines occurring at the level of the brain and spinal cord.

Previous studies indicated that in response to *L. major*, KC mRNA was rapidly and transiently induced in the infected skin (Muller et al., 2001) and that IL-8/CXCL8 (human homologue of KC) or CINC-1/CXCL (rat homolog of KC) induced mechanical hypernociception is inhibited by β–adrenergic receptor antagonists, but not by COX inhibitors (Cunha et al., 1991). However, no studies have been done previously about the levels of KC in the *L. major* evoked hyperalgesia. In this study, the levels of KC increased significantly in the infected paws of mice at days 2 and 7 post-infection as compared to control levels. However, atenolol did not result in any significant change in the levels of this cytokine during the period of treatment (except at day 7 post-infection) while the peripheral hyperalgesia was reduced. These results suggest that although KC might play a role in provoking *L. major* induced peripheral hyperalgesia, it doesn’t contribute neither to sustained hyperalgeisa nor to the atenolol reversal of the low pain thresholds. Furthermore, KC seems to play no role in the central hypernociception since it was not detected in the non-infected paws of mice while the tails flicks threshold values were significantly low.

## Conclusion

Our results provide many new insights about the basic mechanisms involved in the *L. major* induced hyperalgesia.

We demonstrate here that atenolol reduces peripheral hyperalgesia but has no significant effect on central hyperalgesia. This effect seems to be mediated by the reduction of the TNF-α levels in the infected paws of mice. On the other hand, atenolol did not affect the levels of IL-lβ giving further evidence that this cytokine does not play a direct role in peripheral or central hyperalgesia, which is in accordance with previous studies performed by our group (Karam et al., 2011). Furthermore, our results demonstrate that atenolol has no effect on the levels of KC, which seems to play no major role neither in the sustained peripheral hyperalgesia nor in the central one. Taken together, and in addition to the observation that indomethacin does not affect the low pain thresholds in *L. major* infected mice, these results provide further evidence that the sympathetic amines play a more pronounced role in this sustained cutaneous leishmaniasis hyperalgesia model involving a mediator other than KC. Therefore, TNF-α seems to either directly hypersensitize the nociceptors or induces the sympathetic amines which can similarly affect those terminal nerve endings making of the beta blockers a potential efficient treatment for *L. major* induced peripheral hyperalgesia.

## Author Contributions

The authors researched, discussed, and approved the concept, drafted, and submitted the paper. All co-authors made a significant intellectual contribution to the concept and data presented in the manuscript.

## Conflict of Interest Statement

The authors declare that the research was conducted in the absence of any commercial or financial relationships that could be construed as a potential conflict of interest.
